# Stroke Epidemiology, Care, and Outcomes in Kenya: A Scoping Review

**DOI:** 10.3389/fneur.2021.785607

**Published:** 2021-12-16

**Authors:** Peter Waweru, Samwel Maina Gatimu

**Affiliations:** ^1^Department of Neurosurgery, Kenyatta University Teaching, Referral and Research Hospital, Nairobi, Kenya; ^2^Diabetic Foot Foundation of Kenya, Nairobi, Kenya

**Keywords:** stroke, Kenya, sub-Saharan Africa, Africa, East Africa

## Abstract

**Background:** Stroke is a leading cause of death and disability in sub-Saharan Africa with increasing incidence. In Kenya, it is a neglected condition with a paucity of evidence despite its need for urgent care and hefty economic burden. Therefore, we reviewed studies on stroke epidemiology, care, and outcomes in Kenya to highlight existing evidence and gaps on stroke in Kenya.

**Methods:** We reviewed all published studies on epidemiology, care, and outcomes of stroke in Kenya between 1 January 1990 to 31 December 2020 from PubMed, Web of Science, EBSCO*host*, Scopus, and African journal online. We excluded case reports, reviews, and commentaries. We used the Newcastle-Ottawa scale adapted for cross-sectional studies to assess the quality of included studies.

**Results:** Twelve articles were reviewed after excluding 111 duplicates and 94 articles that did not meet the inclusion criteria. Five studies were of low quality, two of medium quality, and five of high quality. All studies were hospital-based and conducted between 2003 and 2017. Of the included studies, six were prospective and five were single-center. Stroke patients in the studies were predominantly female, in their seventh decade with systemic hypertension. The mortality rate ranged from 5 to 27% in-hospital and 23.4 to 26.7% in 1 month.

**Conclusions:** Our study highlights that stroke is a significant problem in Kenya, but current evidence is of low quality and limited in guiding policy development and improving stroke care. There is thus a need for increased investment in hospital- and community-based stroke care and research.

## Introduction

Sub-Saharan Africa (SSA) has the highest stroke burden globally with a steadily increasing incidence estimated at 316 cases per 100,000 persons (1–4). Stroke in SSA occurs in relatively young patients and tends to be severe due to uncontrolled risk factors (5); resulting in high personal and societal costs (6) and significant disability (3).

While significant progress has been made in stroke care in high-income countries, stroke care in SSA is disjointed with glaring gaps in all areas of the stroke care continuum (7). This is due to poor health infrastructure, shortage of specialists, poor health financing models, lack of and poor implementation of health policies and resources, and poor leadership and governance that characterize most health systems in SSA (8). Consequently, specialized stroke care—from prehospital, hyperacute and acute stroke care, to rehabilitation and secondary prevention—is markedly underdeveloped (6) resulting in high morbidity and mortality (9–11).

Stroke in Kenya is a neglected condition despite the urgent care stroke patients need. Most stroke patients are managed in general wards by non-neurologists and clinical officers (the equivalent of physician assistants) with minimal training in stroke care. In addition, stroke patients are usually referred to private diagnostic facilities for neuroimaging due to breakdown and/or lack of scanners, which are only available in 13% of health facilities (12) adding to the financial burden of stroke. These highlight far-reaching gaps in stroke care in Kenya; the extent to which is not well known. Thus, we aimed to collate studies on epidemiology, care, and outcomes of stroke in Kenya to guide further action in stroke research, policy, and care.

## Methods

We conducted a systematic scoping review of all studies reporting on stroke in Kenya. The *Preferred Reporting Items for Systematic Reviews and Meta-Analyses for Scoping Review (PRISMA-SR)* guided the conduct and reporting of this review (Supplementary Table 1).

First, two reviewers performed a comprehensive search in the following databases: EBSCO*host*, PubMed, Web of Science, Scopus, Africa Journal Online (AJOL) and Google Scholar. Reference lists of included studies and Kenyan universities' repositories were also searched. The search terms included “*stroke*” OR “*cerebrovascular accident*” OR “*CVA*” OR “*cerebrovascular event” OR “CVE”* OR “*transient ischemic attack*” OR “*TIA*” OR “*brain hemorrhage*” OR “*hemorrhagic stroke*” OR “*ischemic stroke*” AND “*Kenya*.”

Second, all identified studies were imported to the Endnote (x8) reference manager for removal of duplicates and further review. Third, one reviewer (PW) read the titles and abstracts of records obtained through the electronic searches and excluded irrelevant studies. Fourth, full texts of the remaining studies were obtained, and two reviewers (PW and SMG) read the full text and independently selected studies for inclusion based on the following eligibility criteria:

- **Study design**: Community- and hospital-based observational and experimental studies.
- **Study setting**: Conducted in Kenya.
- **Outcome**: Stroke incidence, prevalence, subtypes, risk factors, recognition and response, imaging and laboratory investigations, thrombolysis or thrombectomy, inpatient care, neurorehabilitation and post-stroke care, secondary prevention, short or long-term outcomes (mortality and functional, psychiatric, and neurocognitive outcomes), quality of life and cost of stroke.
- **Participants:** Adult stroke patients or stroke care providers.
- **Timelines:** 1 January 1990 and 31 December 2020.

We excluded studies with no full text, review articles, case reports and commentaries, and unpublished theses and dissertations.

The reviewers independently assessed the quality of the included studies using the “*Newcastle-Ottawa Quality Assessment Scale”* adapted for cross-sectional studies (13). In cases where the reviewers could not agree, an average quality score was computed.

The two review authors independently extracted data from the included studies using a standard data extraction form and entered it in Microsoft Excel. We compared the extracted data for accuracy and consistency and agreed through discussion on any inconsistencies before summarizing them in the results section (Table 1).

**Table 1:** Characteristics of included studies.

**References**	**Title**	**Year of publication**	**Year(s) of study**	**Design**	**Setting**	**Study population**	**Case definition**	**% of stroke cases confirmed by neuroimaging**	***n***	**Mean age (SD)**	Peak age, years^**^	**Female %**	**HS%**
Oduor et al. (14)	Stroke types, risk factors, quality of care and outcomes at a Referral Hospital in Western, Kenya	2015	2010–2014	Retrospective	Urban/Public	All strokes, ≥18 years	WHO definition of stroke	100	155	61 (49–72)^*^	>69	58	52
Kaduka et al. (15)	Stroke distribution patterns and characteristics in Kenya's leading public health tertiary institutions: Kenyatta National Hospital and Moi Teaching and Referral Hospital	2018	2015–2016	Prospective	Urban/Public	All strokes, ≥18 years	CT/MRI confirmed stroke	100	691	60 (45-73) *	60–69	57.5	44.4
Kaduka et al. (16)	Stroke mortality in Kenya's Public Tertiary Hospitals: a prospective facility-based study	2018	2015–2016	Prospective	Urban/Public	All strokes, ≥18 years	CT/MRI confirmed stroke	100	719	58.6 (18.7)	60–69	56.7	43.9
Kaduka et al. (17)	Disability-adjusted life-years due to stroke in Kenya	2019	2015–2016	Prospective	Urban/Public	All strokes, ≥18 years	CT/MR confirmed stroke	100	719	58.6 (18.7)	60–64	56.7	43.9
Ominde et al. (18)	Pattern of stroke in a rural Kenyan hospital	2019	2015–2016	Prospective	Rural/Public	All strokes, Adults	WHO definition of stroke	100	227	68.8 (6.8)	60–69	62	32.6
Jowi et al. (19)	Pathological sub-types, risk factors and outcome of stroke at the Nairobi Hospital, Kenya	2009	2003–2006	Retrospective	Urban/ Private	All strokes, ≥18 years	WHO definition of stroke	100	80	61.3	–	46.2	8.8
Muli et al. (20)	Quality of life amongst young adults with stroke living in Kenya	2013	2007–2008	Retrospective	Urban/ Public	15–49 years	Not defined	–	161	–	40–49	55.9	–
Ogengo and Olabu (21)	Ischemic cortical stroke in a Kenyan Referral Hospital	2015	2007–2011	Retrospective	Urban/ Public	Cortical, ischemic strokes, ≥18 years	Paralysis, aphasia, and headache combined with CT scan and angiographic findings.	100	377	54.72 (16.8)	–	54.6	–
Ogolla and Opemo (22)	Early mobilization and physical activity improve stroke recovery: a cohort study of stroke inpatients in Kisumu County Referral Hospitals, Kenya	2016	2015	Prospective	Urban	All strokes, ≥18 years	Not defined	–	100	59.1 (2.3)	–	61	–
Wanjiru Kingau (23)	Care process for stroke patients in Kenya: mixed study	2018	2014	Retrospective	Urban–Rural/ Public	All strokes, Adults	Not defined	–	150	61.7 (16.7)	–	36.5	–
Wairoto et al. (24)	Prevalence and nature of psychiatric morbidity in stroke outpatients in Kenyatta national hospital, Kenya	2020	2015	Prospective	Urban	All strokes, ≥18 years	Not defined	–	210	–	–	41	–
Waweru and Gatimu (25)	Mortality and functional outcomes after a spontaneous subarachnoid hemorrhage: A retrospective multicenter cross-sectional study in Kenya	2019	2009–2017	Retrospective	Urban	SAH, ≥18 years	Suggestive presentation supported by computed tomography, lumbar puncture, or necropsy evidence of SAH	100	158	48.6 (15.9)	–	57.6	All

*HS, haemorrhagic stroke; SAH, subarachnoid hemorrhage*.

* *Median (IQR);*

** *Range*.

## Results

**Figure 1:**
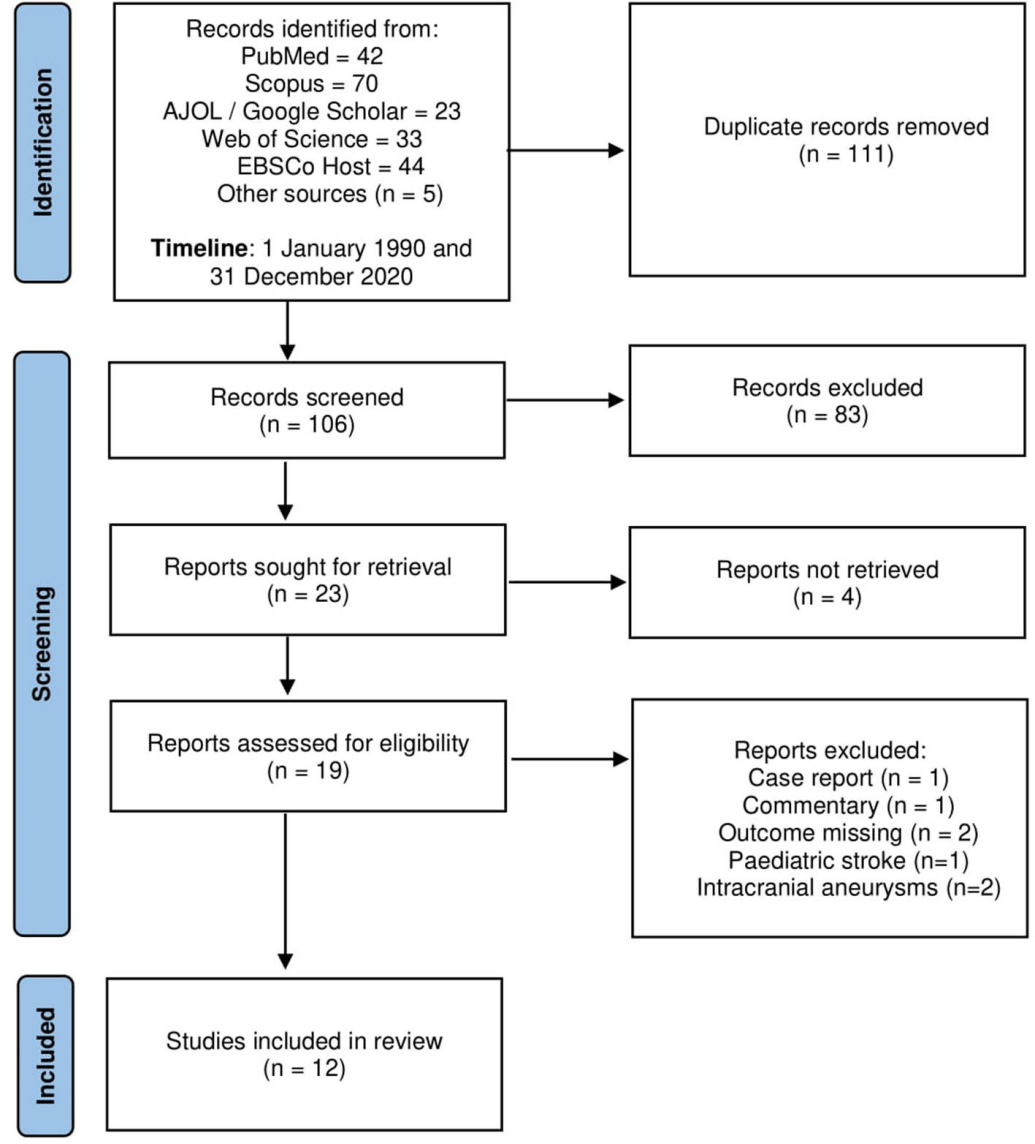
PRISMA flow chart of selected studies.

Of the 217 articles identified from a database search as well as a search of grey literature, 111 duplicates were removed. Nineteen full texts were screened for eligibility and 12 articles were included (Figure 1).

### Characteristics of Included Studies

Table 1 highlights the characteristics of the included studies. Twelve hospital-based studies conducted between 2003 and 2017 met the inclusion criteria (14–25). Six studies were retrospective (14, 19–21, 23, 25) while six were prospective (15–18, 22, 24). One study was conducted in a rural hospital (18), and one in a private hospital (19). Five studies were single-center (14, 18, 19, 21, 24) while seven were multicenter (15–17, 20, 22, 23, 25). One study included only cortical ischemic stroke patients (21) while one other study included only patients with chronic (over two months) strokes aged 15–49 years (20). Only one study on subarachnoid hemorrhage patients was included (25).

### Quality Assessment of Included Studies

Five studies were of low quality, two of medium quality and five of high quality (Supplementary Table 2). Six studies were not representative, five had poorly calculated or unexplained sample sizes, seven studies did not control for confounders and seven studies did not perform the appropriate statistical tests.

### Epidemiology of Stroke in Kenya

The incidence and prevalence of stroke in Kenya remain largely unknown. There was a varied point prevalence of stroke in three hospital-based studies: 0.6% in a referral hospital in Western Kenya (14), 3.0% in an urban private hospital in Nairobi (19) and 7.1% in a rural hospital in Eastern Kenya (18). Most stroke cases were ischemic (48–85%), with the proportion of hemorrhagic strokes ranging from 8.8 to 52% (14–16, 19). The rate of stroke recurrence was 15% in two studies based in tertiary referral centers (14, 15) and 20% in a rural hospital-based study (18).

Overall, stroke peaked in the seventh decade (15, 16, 18) with a mean age of 58.6 in a prospective multicenter referral hospital-based study (16), 61.3 years in a Nairobi-based private hospital (19) and 68.8 years in a rural hospital (18). There was a female predominance among stroke patients in most studies with the highest proportion of female stroke patients being in the rural hospital (62%) (18). Besides age and sex, other major risk factors of stroke included hypertension (50–80%), diabetes (4–32.5%) and human immunodeficiency virus (HIV) infection (2.5–12%) (Table 2).

**Table 2:** Risk factors and outcomes of stroke in Kenya.

**References**	**n**	**Risk factors**							**In-hospital deaths %**	**Dead/Lost to follow-up (%)^§^**				
		**HTN %**	**DM%**	**Smoking %**	**Alcohol %**	**Previous stroke %**	**Hypercholesterolemia %**	**HIV %**		**10 days**	**28-days**	**90 days**	**180 days**	**1 year**
Jowi and Mativo (19)	80	80	32.5	–	–	–	36.5^*^	2.5	5	–	–	–	–	–
Kaduka et al. (15)	691	77.3	14.9	16.1		15.4	2.8^**^	8		16.4 (4.2)	23.4 (7.8)	29.8 (21)	32 (38.2)	
Ominde et al. (18)	227	74	32	48	63	20	–	12	–	–	–	–	–	–
Oduor et al. (14)	155	73	4	9	24	15	84^†^	10	27	–	–	–	–	–
Kaduka et al. (16)	719	–	–	–	–	–	–	–	21.6	18.4 (1.3)	26.7 (1.7)	34.1 (22.1)	37.8 (22.9)	41.7 (26.1)
Waweru and Gatimu (25)	158	50	–	14.2	25.4	–	–	–	24.1	–	26.6	–	–	–

* *LDL ≥ 3.36 ml/l;*

** *HDL-C <1.3 mmol/l; triglycerides > 2.2 mmol/l; total cholesterol > 6.2 mmol/l; low-density lipoprotein cholesterol > 4.1 mmol/l, or specific treatment for hypercholesterolemia*.

† *Elevated plasma total cholesterol levels of >5.18 mmol/l or LDL cholesterol levels >2.6 mmol/l or HDL levels <1.3 mmol/l, or had been using lipid-lowering medication*.

§ *Percentage of patients lost to follow-up in brackets; HTN, hypertension; DM, diabetes mellitus*.

SAH was commonest in females (58%) with a mean age of 48.6 (SD 15.9); a majority (62%) being aneurysmal SAH (aSAH) although only 58% of all patients had cerebral angiography (25).

### Stroke Care and Outcomes

Very limited data was found on the quality and access of stroke care services in the country. Only one study (14) described quality parameters for stroke care including the time from stroke onset to a presentation [median: 2 days (IQR: 1–3 days)], stroke severity assessment (92% by Glasgow coma scale), provision of antithrombotic within 48 h (73%), ambulation within 48 h (33%), thromboprophylaxis (24%), smoking cessation counseling (25%) and dysphagia screening before oral intake (3%). Vascular imaging for ischemic stroke via carotid doppler imaging is more common in private/urban hospitals, but even then was only 40% in a private hospital study (19). Even less data was available on stroke rehabilitation (22) with only one low-quality study reporting on inpatient physiotherapy (23). Secondary prevention was also very basic with only two-thirds of patients discharged on antithrombotic drugs (14).

In-hospital mortality in public hospitals ranged from 21.6 to 27% (14, 16) while it was only 5% in a private hospital (19). About a quarter of stroke patients died in 1 month (23.4–26.7%); and about a third (29.8–34.1%) in 3 months (15, 16). Stroke mortality was associated with age (higher in older patients), sex (higher in females) and stroke type (higher in ischemic stroke overall at 12 months but higher in hemorrhagic stroke at 1 month) (16). In aSAH patients, only half of the patients had surgical or endovascular management, and this was delayed for up to 20 days after presentation, especially in public hospitals. Consequently, a quarter of patients with SAH died in hospitals, and less than half (46%) had favorable functional outcomes on follow-up (25).

No studies described cognitive outcomes. One low-quality study reported on the psychiatric outcomes (24); with a third of stroke patients having some psychiatric morbidity; majorly depression (19%) and general anxiety disorder (9.5%).

## Discussion

Evidently, little is known about stroke in Kenya. Current literature on stroke epidemiology, stroke care and post-stroke outcomes in Kenya is of low-medium quality. From current evidence, stroke in Kenya is of unknown incidence and prevalence, largely affecting patients in their sixth and seventh decades; with an even earlier onset in subarachnoid hemorrhage stroke subtypes; and predominantly affecting female patients, with hypertension being a major risk factor. After a stroke, care is mainly basic and unsurprisingly, outcomes are poor.

In other sub-Saharan Africa studies such as the INTERSTROKE study (5) similar findings have been reported, including the predominance of female stroke patients, younger stroke patients compared to other regions, a higher proportion of hemorrhagic strokes and poorer outcomes.

These findings reflect the neglect stroke patients face in sub-Saharan Africa due to a lack of resources. The female predominance of stroke patients in the region is likely attributable to the hospital-based nature of most of the regional studies. This is further impacted by the fact that most men in the region have poor health-seeking behaviors due to financial incapacity (26), as well as the higher stroke severity in women (27) which would lead to most men with relatively minor strokes opting for alternative therapies that are not uncommon in the region.

In Kenya, primary stroke prevention is underfunded and of limited reach, symbolic of primary health care systems in the region. Consequently, while about a quarter of the Kenyan population is hypertensive (28), only 22.3% are on treatment and only 3% have controlled hypertension (29). The bulk of resultant uncontrolled hypertension has been cited as a plausible reason for the higher proportion of hemorrhagic stroke in the region (29–60%) compared to Western countries (16–20%) (3, 5, 30, 31). The difference in the proportion of stroke subtypes has also been attributed to the tendency to scan only stroke patients with more severe strokes in this resource-limited setting, of which hemorrhagic strokes predominate (4).

After a stroke, Kenyan and sub-Saharan Africa stroke patients are faced with many hurdles including delayed transportation due to lack of emergency response systems, ill-equipped hospitals, and lack of stroke specialists (11, 32–34). In Kenya, for instance, only 13% of health facilities have computed tomography scanners which are so central in stroke diagnosis; and only half of the health facilities have aspirin (12). Worse, only 4% of health facilities in Kenya offer rehabilitation services, largely physiotherapy with limited speech and occupation therapy (12). Furthermore, Kenya has only eight registered adult neurologists (35) against a population of 50 million persons and a growing burden of stroke. Most of these specialists are in large urban or private hospitals where resources for stroke care are less limited. However, to improve stroke care in Kenya, the government has recently through the Managed Equipment Services equipped most national and county referral hospitals with imaging equipment such as CT scans (36). There is also increased sensitization and enrolment of the population to the National Health Insurance Fund, as a pathway to access to universal healthcare and to reduce the catastrophic health expenditure incurred by patients including stroke patients in the country. Concurrently, the county governments—which oversee health service delivery—have continued to increase investment in the health sector to provide accessible and quality health care. Despite these efforts, stroke is still not a prioritized non-communicable disease and the country lacks clear national stroke prevention, treatment and rehabilitation strategy or policies. For example, the current non-communicable disease strategy 2021–2025 only tracks an increase in the coverage of hypertensive and diabetes patients receiving drug therapy or counseling from 6.2 to 50% by 2025 (37).

Unsurprisingly, while about 5% of stroke patients died in a private hospital (19), this was 4-fold in public hospitals (15, 16). Such high mortality post-stroke in SSA has been reported with <50% of patients alive at 12 months (30). Similar to our study, about a quarter of stroke patients die within 1 month in the region (38), which is about five times more deaths than in Western countries (5).

Overall, stroke care in Kenya remains suboptimal due to the lack of clear and coordinated stroke prevention and management policies and models of stroke care. However, with the recent focus on universal healthcare, the country could learn and adopt some of the best practices on stroke prevention and models of care from other low resource settings. One such model—the China Stroke Prevention Project Committee (CSPPC) program—aimed to establish prevention and control systems for stroke in China (39). The program improved primary prevention strategies, established stroke centers for the treatment of stroke patients—organized into advanced stroke centers and stroke prevention centers—and established stroke maps for different cities to define timely transport of stroke patients and ensure early treatment (39). A similar model in Kenya would entail stratifying stroke care into basic (primary) stroke care centers tasked with stroke prevention, basic care and neurorehabilitation and advanced/comprehensive stroke centers with capacity of thrombolysis, thrombectomy and neurosurgery. The model could easily be implemented within Kenya's hierarchical health care system; with the county (level 5) and national (level 6) referral hospitals tasked with comprehensive stroke care and lower levels (levels 1–4) tasked with a timely referral of patients, patient follow-up, primary and secondary prevention, including community-based neurorehabilitation. Similarly, transit of patients can be streamlined using a similar “green map” as has been utilized by the CSPPC model in China though the economic models that would allow such a realignment of stroke care and establishment of stroke care models need further research. Moreover, patients' organizations such as the Stroke Association of Kenya could play a pivotal role in creating awareness on stroke and advocating for better models of stroke care in Kenya.

## Limitations

This review maps out and assesses the quality of current evidence on stroke in Kenya using a systematic approach. The study, however, has some limitations. First, only 12 studies met the inclusion criteria of the review; five of which were of low quality. Second, the studies included may have underestimated the burden of stroke in Kenya due to: (i) potential referral bias in hospital-based studies, (ii) incomplete data in retrospective studies and (iii) lack of representativeness in hospital-based and single-centered studies. Only one study was conducted in a rural setting despite two-thirds of the population in Kenya living in rural areas. Third, most studies did not report on aspects of stroke care including hyperacute, inpatient and rehabilitation care, secondary prevention, and functional, neurocognitive, and psychiatric outcomes limiting a better understanding of stroke in Kenya.

## Conclusion

This study clearly depicts a dearth of evidence on stroke in Kenya, a trend evident in other SSA countries. Similar to other studies in the region, stroke is common in females, occurs at a relatively younger age and has poor outcomes owing to lack of quality care, especially among patients in public hospitals. Current evidence is limited in guiding policy development and improving stroke care in Kenya. There is a need for increased investment in hospital- and community-based stroke care and research. Specifically, more studies focused on the epidemiology of stroke, stroke knowledge, recognition, and emergency response, hyperacute and acute care, post-stroke care, quality and cost of stroke care, and stroke outcomes are still needed.

## Author Contributions

PW and SG contributed equally to the conceptualization, study design, data extraction, analysis, and interpretation of the study findings. All authors critically reviewed, read, and approved the manuscript.

## Conflict of Interest

The authors declare that the research was conducted in the absence of any commercial or financial relationships that could be construed as a potential conflict of interest.

## Publisher's Note

All claims expressed in this article are solely those of the authors and do not necessarily represent those of their affiliated organizations, or those of the publisher, the editors and the reviewers. Any product that may be evaluated in this article, or claim that may be made by its manufacturer, is not guaranteed or endorsed by the publisher.

## References

[1] Boutayeb A Derouich M Boutayeb W Lamlili MEN. Cerebrovascular diseases and associated risk factors in WHO Eastern mediterranean countries. Cardiol Angiol. (2014) 2, 62–75. 10.9734/CA/2014/9731

[2] Lekoubou A Nkoke C Dzudie A Kengne AP. Stroke admission and case-fatality in an urban medical unit in sub-Saharan Africa: a fourteen year trend study from 1999 to 2012. J Neurol Sci. (2015) 350:24–32. 10.1016/j.jns.2015.02.002 25684340

[3] Owolabi MO Akarolo-Anthony S Akinyemi R Arnett D Gebregziabher M Jenkins C. The burden of stroke in Africa: a glance at the present and a glimpse into the future. Cardiovasc J Afr. (2015) 26(2 Suppl 1):S27–38. 10.5830/CVJA-2015-038 25962945 PMC4557491

[4] Walker RW Viney R Green L Mawanswila M Maro VP Gjertsen C. Trends in stroke admissions to a Tanzanian hospital over four decades: a retrospective audit. Trop Med Int Health. (2015) 20:1290–6. 10.1111/tmi.12547 25983015

[5] O'Donnell MJ Xavier D Liu L Zhang H Chin SL Rao-Melacini P. Risk factors for ischaemic and intracerebral haemorrhagic stroke in 22 countries (the INTERSTROKE study): a case-control study. Lancet. (2010) 376:112–23. 10.1016/S0140-6736(10)60834-3 20561675

[6] Ekeh BC. Challenges of the management of stroke in Sub Saharan Africa: evaluating awareness, access and action. J Pediatr Neurol Med. (2017) 02:128. 10.4172/2472-100x.1000128

[7] Campbell BC Meretoja A Donnan GA Davis SM. Twenty-year history of the evolution of stroke thrombolysis with intravenous alteplase to reduce long-term disability. Stroke. (2015) 46:2341–6. 10.1161/STROKEAHA.114.007564 26152294

[8] Oleribe OO Momoh J Uzochukwu BS Mbofana F Adebiyi A Barbera T. Identifying key challenges facing healthcare systems in africa and potential solutions. Int J Gen Med. (2019) 12:395–403. 10.2147/IJGM.S223882 31819592 PMC6844097

[9] Nakibuuka J Sajatovic M Nankabirwa J Ssendikadiwa C Furlan AJ Katabira E. Early mortality and functional outcome after acute stroke in Uganda: prospective study with 30 day follow-up. Springerplus. (2015) 4:450. 10.1186/s40064-015-1252-8 26322256 PMC4547979

[10] Baatiema L Chan CKY Sav A Somerset S. Interventions for acute stroke management in Africa: a systematic review of the evidence. Syst Rev. (2017) 6:213. 10.1186/s13643-017-0594-4 29065915 PMC5655819

[11] Urimubenshi G Cadilhac DA Kagwiza JN Wu O Langhorne P. Stroke care in Africa: a systematic review of the literature. Int J Stroke. (2018) 13:797–805. 10.1177/1747493018772747 29664359

[12] Ministry of Health. Kenya Harmonized Health Facility Assessment (KHFA), 2018/2019. Nairobi: Ministry of Health (2020).

[13] Herzog R Álvarez-Pasquin MJ Díaz C Del Barrio JL Estrada JM Gil Á. Are healthcare workers' intentions to vaccinate related to their knowledge, beliefs and attitudes? A systematic review. BMC public health. (2013) 13:1–17. 10.1186/1471-2458-13-154 23421987 PMC3602084

[14] Odour C Keter A Diero L Siika A Williams L. Stroke types, risk factors, quality of care and outcomes at a referral hospital in Western Kenya. East Afr Med J. (2015) 92:324–32.

[15] Kaduka L Korir A Oduor CO Kwasa J Mbui J Wabwire S. Stroke distribution patterns and characteristics in Kenya's leading public health tertiary institutions: Kenyatta National Hospital and Moi Teaching and Referral Hospital. Cardiovasc J Afr. (2018) 29:68–72. 10.5830/CVJA-2017-046 29745965 PMC6008906

[16] Kaduka L Muniu E Oduor C Mbui J Gakunga R Kwasa J. Stroke mortality in Kenya's public tertiary hospitals: a prospective facility-based study. Cerebrovasc Dis Extra. (2018) 8:70–9. 10.1159/000488205 29895000 PMC6031945

[17] Kaduka L Muniu E Mbui J Oduor Owuor C Gakunga R Kwasa J. Disability-adjusted life-years due to stroke in Kenya. Neuroepidemiology. (2019) 53:48–54. 10.1159/000498970 30986786

[18] Ominde BS Ogeng'o JA Misiani MK Kariuki BN. Pattern of stroke in a rural Kenyan hospital. Malawi Med J. (2019) 31, 50–55. 10.4314/mmj.v31i1.9 31143397 PMC6526339

[19] Jowi JO Mativo PM. Pathological sub-types, risk factors and outcome of stroke at the Nairobi Hospital, Kenya. East Afr Med J. (2008) 85:572–81. 10.4314/eamj.v85i12.43535 19413212

[20] Muli G Rhoda A. Quality of life amongst young adults with stroke living in Kenya. Afr Health Sci. (2013) 13:632–8. 10.4314/ahs.v13i3.16 24250300 PMC3824418

[21] Ogengo JA Olabu BO. Ischemic cortical stroke in a Kenyan referral hospital. J Mol Biomark Diag. (2015) 06:1–4. 10.4172/2155-9929.1000238

[22] Ogolla MM Opemo DO. Early mobilization and physical activity improve stroke recovery: a cohort study of stroke inpatients in Kisumu County referral hospitals, Kenya. Am J Public Health Res. (2016) 4:154–8. 10.12691/ajphr-4-4-6

[23] Wanjiru Kingau N. Care process for stroke patients in kenya: mixed study. Health Educ Care. (2018) 3:136. 10.15761/hec.1000136

[24] Wairoto DK Joseph OC Kigamwa PA. Prevalence and nature of psychiatric morbidity in stroke outpatients in Kenyatta national hospital, Kenya. Int J Adv Multidiscip Res. (2020) 7:27–39. 10.22192/ijamr.2020.07.01.004

[25] Waweru P Gatimu SM. Mortality and functional outcomes after a spontaneous subarachnoid haemorrhage: a retrospective multicentre cross-sectional study in Kenya. PLoS ONE. (2019) 14:e0217832. 10.1371/journal.pone.0217832 31188844 PMC6561561

[26] Ministry of Health. 2013 Kenya Household Health Expenditure and Utilisation Survey. Nairobi: Government of Kenya (2014).

[27] Girijala RL Sohrabji F Bush RL. Sex differences in stroke: review of current knowledge and evidence. Vasc Med. (2017) 22:135–45. 10.1177/1358863X16668263 27815349

[28] Gatimu SM John TW. Socioeconomic inequalities in hypertension in Kenya: a decomposition analysis of 2015 Kenya STEPwise survey on non-communicable diseases risk factors. Int J Equity Health. (2020) 19:213. 10.1186/s12939-020-01321-1 33267846 PMC7709247

[29] MoH KNBS WHO. Kenya STEPwise Survey for Non-Communicable Diseases Risk Factors 2015 Report. First edition. Nairobi, Kenya: Ministry of Health, Kenya National Bureau of Statistics and World Health Organization (2015).

[30] Kengne AP Anderson CS. The neglected burden of stroke in Sub-Saharan Africa. Int J Stroke. (2006) 1:180–90. 10.1111/j.1747-4949.2006.00064.x 18706015

[31] Keates AK Mocumbi AO Ntsekhe M Sliwa K Stewart S. Cardiovascular disease in Africa: epidemiological profile and challenges. Nat Rev Cardiol. (2017) 14:273–93. 10.1038/nrcardio.2017.19 28230175

[32] Walker RW Jusabani A Aris E Gray WK Mitra D Swai M. A prospective study of stroke sub-type from within an incident population in Tanzania. S Afr Med J. (2011) 101:338–44. 10.7196/samj.4511 21837879

[33] Goldstein LB. Stroke in sub-Saharan Africa: an urgent call for prevention. Neurology. (2013) 81:403–04. 10.1212/01.wnl.0000432935.60352.a4 23877799

[34] Chin JH. Stroke in sub-Saharan Africa: an urgent call for prevention. Neurology. (2012) 78:1007–8. 10.1212/WNL.0b013e318248df95 22454267

[35] Kenya Medical Practitioners Dentists Council. Licenced Local Specialist Practitioners for the year 2021 as at 23/02/2021 [Online]. Nairobi: Kenya Medical Practitioners and Dentists Council (2021). Available online at: http://kmpdc.go.ke/Registers/Specialist_Practitioners.php (accessed February 22, 2021).

[36] Mutua J Wamalwa N. Leasing of Medical Equipment Project in Kenya: Value for Money Assessment. Africa Portal (2020).

[37] Ministry of Health. Kenya National Strategy for the Prevention and Control of Non-Communicable Diseases (NCDs) 2021–2025. Nairobi, Kenya: Ministry of Health, Kenya (2021).

[38] Walker RW Jusabani A Aris E Gray WK Whiting D Kabadi G. Post-stroke case fatality within an incident population in rural Tanzania. J Neurol Neurosurg Psychiatry. (2011) 82:1001–5. 10.1136/jnnp.2010.231944 21386108

[39] Chao B-H Yan F Hua Y Liu J-M Yang Y Ji X-M. Stroke prevention and control system in China: CSPPC-Stroke Program. Int J Stroke. (2021) 16:265–72. 10.1177/1747493020913557 32223541

