# Supplementary material for: Stroke Epidemiology, Care, and Outcomes in Kenya: A Scoping Review
Source: Front Neurol. 2021 Dec 16;12:785607. doi: 10.3389/fneur.2021.785607 (PMC8716633; doi:10.3389/fneur.2021.785607)
Supplement: Supplementary file 2 [file Table_2.docx]

**Supplementary Table 2. Newcastle-Ottawa quality assessment scale for cross-sectional studies**

|  |  | **Selection** | | | | **Comparability** | **Outcome** | |  |
| --- | --- | --- | --- | --- | --- | --- | --- | --- | --- |
| **Source** | **Title** | **Representativeness of**  **the Sample** | **Sample**  **Size** | **Non-**  **Respondents** | **Ascertainment**  **of the**  **Exposure** | **The Subjects in**  **Different**  **Outcome Groups**  **are Comparable (Confounding)** | **Assessment**  **of the**  **Outcome** | **Statistical test** | **Quality** |
| Jowi 2008 ([Jowi and Mativo, 2008](#_ENREF_10)) | Pathological sub-types, risk factors and outcome of stroke at the Nairobi Hospital, Kenya | - | * | * | ** | - | ** | - | Medium |
| Muli 2013 ([Muli and Rhoda, 2013](#_ENREF_23)) | Quality of life amongst young adults with stroke living in Kenya | - | - | - | - | - | * | * | Low |
| Oduor 2015 ([Odour et al., 2015](#_ENREF_27)) | Stroke types, risk factors, quality of care and outcomes at a Referral Hospital in Western, Kenya | * | * | * | ** | - | ** | * | High |
| Ominde 2019 ([Ominde et al., 2019](#_ENREF_31)) | Pattern of stroke in a rural Kenyan hospital | * | * | * | ** | - | - | - | Medium |
| Ogeng’o 2015 ([Ogeng o and Olabu, 2015](#_ENREF_28)) | Ischemic Cortical Stroke in a Kenyan Referral Hospital | - | * | - | ** | * | - | - | Low |
| Ogolla 2015 ([Ogolla and Opemo, 2016](#_ENREF_29)) | Early Mobilization and Physical Activity Improve Stroke Recovery: A Cohort Study of Stroke Inpatients in Kisumu County Referral Hospitals, Kenya | - | - | - | - | - | * | - | Low |
| *Kaduka 2018 (*[*Kaduka et al., 2018a*](#_ENREF_11)*)* | *Stroke distribution patterns and characteristics in Kenya’s leading public health tertiary institutions: Kenyatta National Hospital and Moi Teaching and Referral Hospital* | *** | *** | *** | **** | **** | **** | *** | *High* |
| *Kaduka 2018 (*[*Kaduka et al., 2018b*](#_ENREF_14)*)* | *Stroke Mortality in Kenya’s Public Tertiary Hospitals: A Prospective Facility-Based Study* | *** | *** | *** | **** | **** | **** | *** | *High* |
| *Kaduka 2019 (*[*Kaduka et al., 2019b*](#_ENREF_13)*)* | *Disability-Adjusted Life-Years Due to Stroke in Kenya* | *** | *** | *** | **** | **** | **** | *** | *High* |
| Kingau 2018 ([Wanjiru Kingau, 2018](#_ENREF_38)) | Care process for stroke patients in Kenya: mixed study | - | - | - | * | - | - | - | Low |
| Wairoto 2020 ([Wairoto et al., 2020](#_ENREF_34)) | Prevalence and nature of psychiatric morbidity in stroke outpatients in Kenyatta national hospital, Kenya | - | - | - | - | * | * | - | Low |
| Waweru 2019 ([Waweru and Gatimu, 2019](#_ENREF_39)) | Mortality and functional outcomes after a spontaneous subarachnoid haemorrhage: A retrospective multicentre cross-sectional study in Kenya | * | * | * | ** | ** | ** | * | High |

**“Selection:** (Maximum 5 stars): (1) Representativeness of the sample: a) Truly representative of the average in the target population. * (all subjects or random sampling); b) Somewhat representative of the average in the target population. * (non-random sampling); c) Selected group of users; d) No description of the sampling strategy. (2) Sample size: a) Justified and satisfactory. *; b) Not justified.; (3) non-respondents: a) Comparability between respondents and non-respondents characteristics is established, and the response rate is satisfactory. *; b) The response rate is unsatisfactory, or the comparability between respondents and non-respondents are unsatisfactory; c) No description of the response rate or the characteristics of the responders and the non-responders. (4) Ascertainment of the exposure (risk factor): a) Validated measurement tool. **; b) Non-validated measurement tool, but the tool is available or described.*; c) No description of the measurement tool. **Comparability:** (Maximum 2 stars): (1) The subjects in different outcome groups are comparable, based on the study design or analysis. Confounding factors are controlled: a) The study controls for the most important factor (select one). *; b) The study control for any additional factor. * **Outcome:** (Maximum 3 stars): (1) Assessment of the outcome: a) Independent blind assessment. **; b) record linkage. **; c) Self-report. *: d) No description. (2) Statistical test: a) The statistical test used to analyze the data is clearly described and appropriate, and the measurement of the association is presented, including confidence intervals and the probability level (p-value). *; b) The statistical test is not appropriate, not described or incomplete*.” (Adapted Newcastle-Ottawa quality assessment scale for cross-sectional studies)*
